# Ibuprofen enantiomers in premature neonates with patent ductus arteriosus: Preliminary data on an unexpected pharmacokinetic profile of *S*(+)‐ibuprofen

**DOI:** 10.1002/chir.23308

**Published:** 2021-03-28

**Authors:** Roberto Padrini, Caterina Ancora, Daniel Nardo, Giovanni De Rosa, Sabrina Salvadori, Luca Bonadies, Anna Chiara Frigo, Paola Lago

**Affiliations:** ^1^ Clinical Pharmacology Unit, Department of Medicine University of Padova Padova Italy; ^2^ Neonatal Intensive Care Unit, Department of Women's and Children's Health University of Padova Padova Italy; ^3^ Biostatistics, Epidemiology and Public Health Unit, Department of Cardiac‐Thoracic‐Vascular Sciences and Public Health University of Padova Padova Italy; ^4^ Neonatal Intensive Care Unit, Women's and Children's Department Cà Foncello Hospital Treviso Italy

**Keywords:** chiral inversion, enantiomer, ibuprofen, plasma levels, preterm infants

## Abstract

*S*(+)‐ibuprofen (*S*‐IBU) and *R*(−)‐ibuprofen (*R*‐IBU) concentrations were measured in 16 neonates with patent ductus arteriosus during a cycle of therapy (three intravenous doses of 10–5–5 mg kg^−1^ at 24‐h intervals), at the end of the first infusion and 6, 24, 48, and 72 h later. Data were analyzed with a PK model that included enantiomer elimination rate constants and the *R*‐ to *S*‐IBU conversion rate constant. The *T*½ of *S*‐IBU in the newborn was much longer than in adults (41.8 vs. ≈2 h), whereas the *T*½ of *R*‐IBU appeared to be the same (2.3 h). The mean fraction of *R*‐ to *S*‐IBU conversion was much the same as in adults (0.41 vs. ≈0.60). *S*‐IBU concentrations measured 6 h after the first dose were higher than at the end of the infusion in 10 out of 16 cases, and in five cases, they remained higher even after 24 h. This behavior is unprecedented and may be attributable to a rapid *R*‐to‐*S* conversion overlapping with a slow *S*‐IBU elimination rate. In 13 of the 16 neonates, *S*‐IBU concentrations at 48 and/or 72 h were lower than expected, probably due to the rapid postnatal maturation of the newborn's liver metabolism.

## Graphical abstract

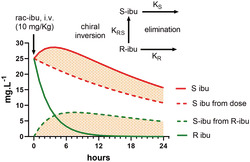

## 1 INTRODUCTION

Racemic ibuprofen (rac‐IBU) is currently used to induce the closure of a patent ductus arteriosus (PDA) in preterm infants by reducing the biosynthesis of vasodilating prostaglandins. Compared with indomethacin, ibuprofen (IBU) exhibits a similar effectiveness with fewer serious adverse effects (necrotizing enterocolitis and transient renal insufficiency), so it appears to be the drug of choice nowadays.^1^ Several authors have studied IBU pharmacokinetics in premature infants after both intravenous and oral administration, albeit using different methodological approaches. Some measured total plasma concentrations of the rac‐IBU mixture.^2^, ^3^, ^4^, ^5^, ^6^ Others separately analyzed the two enantiomers, *S*(+)‐ibuprofen (*S*‐IBU) and *R*(−)‐ibuprofen (*R*‐IBU), after rac‐IBU administration.^7^, ^8^ Measuring plasma levels of both enantiomers is much more informative from a clinical viewpoint because *R*‐IBU is far less active than *S*‐IBU and is converted in vivo into *S*‐IBU by unidirectional chiral inversion.^9^, ^10^

Another methodological difference between previous studies concerns the type of pharmacokinetic analysis conducted. Some authors collected multiple blood samples from the same infant and calculated individual PK parameters.^2^, ^3^, ^6^ Others used sparse blood samples from various infants and performed population PK analyses.^4^, ^7^, ^8^ All except one used a one‐compartment model to describe the IBU concentration profile, and only one study estimated the percentage of chiral inversion from *R*‐IBU to *S*‐IBU.^8^

In general, all authors agreed that rac‐IBU (or *S*‐IBU specifically) has a substantially longer half‐life and slower plasma clearance in preterm neonates than in adults. Interestingly, Gregoire et al. proposed a population PK model for rac‐IBU intravenous administration that included an *R*‐ to *S*‐IBU bioconversion rate constant and distinct rate constants for *S*‐ and *R*‐IBU elimination (Figure 2).^8^

Here, we present some results of the “PARIDA” study (Paracetamol vs. Ibuprofen for Ductus Arteriosus closure in preterm infants), limited to the PK analysis of IBU enantiomers during a therapeutic cycle. The reason for reporting our IBU data in advance lies in our finding an unexpected time course of *S*‐IBU concentrations after intravenous administration. These data are to be considered preliminary.

## 2 METHODS

### 2.1 Study design

The PARIDA study (https://clinicaltrials.gov/ct2/show/NCT02056223; Eudract No. 2013‐004955‐19) was planned to compare the efficacy of rac‐IBU and paracetamol in promoting the closure of PDA in preterm neonates. Inclusion criteria were (1) preterm neonates with ≤32 weeks gestational age; (2) ≤ 72 h of life; (3) diagnosis of hemodynamically significant PDA; and (4) parental written informed consent. The protocol was approved by the Ethics Committee of the “Policlinico‐Azienda Ospedaliera di Padova” (Protocol No. 3114/09/2014).

The two treatments were randomly assigned and the outcomes were assessed by personnel not informed about which treatment was administered. Both drugs were infused intravenously with a syringe pump (2 ml, in 15 min), according the following schedules:

1. paracetamol: 15 mg kg^−1^ every 6 h for 3 days, at 12:00, 18:00, 24:00, and 6:00 h;
2. rac‐IBU: three administrations of 10–5–5 mg kg^−1^ at 24‐h intervals, starting at 12:00; to ensure assessor blindness, a 5% dextrose solution was also infused at 18:00, 24:00, and 6:00 h.

Blood samples (0.5 ml, with EDTA as anticoagulant) were taken from a catheter placed in the umbilical artery at the end of the first infusion (Time 0) and then 6, 24, 48, and 72 h later. Plasma was obtained by centrifugation and stored at −20°C until assayed.

#### 2.1.1 IBU enantiomer assay

One hundred microliters of internal standard solution (rac‐flurbiprofen, 0.1 mg ml^−1^ in methanol) was added to 100 μl of plasma. The sample was acidified with 100 μl of HCl 1 N and extracted with 5‐ml *n*‐hexane in a rotating agitator for 10 min. After centrifugation, the organic phase was transferred into conic tubes and evaporated to dryness at 30°C under a gentle nitrogen stream. The residue was solubilized in 500 μl of mobile phase (see below), and 50 μl was injected into a chiral chromatographic column (Phenomenex Lux, 5‐μm Cellulose‐3, 150 × 4.6 mm) through a Waters 717 Plus autosampler. The mobile phase consisted of a mixture (v/v) of methanol (80%) and 1% formic acid solution (20%), flow rate 1 ml min^−1^ (Waters 1515 isocratic pump). The effluent was analyzed with a UV detector (mod. 2487, Waters) set at 220 nm, connected with the Empower software (Waters) to record and analyze the signal. The calibration curves for *S*‐ and *R*‐IBU were generated by adding increasing volumes of a rac‐IBU solution (0.1 mg ml^−1^ in methanol) to 100 μl of pooled human plasma, to obtain concentrations in the range 5–30 mg L^−1^.

**FIGURE 1:**
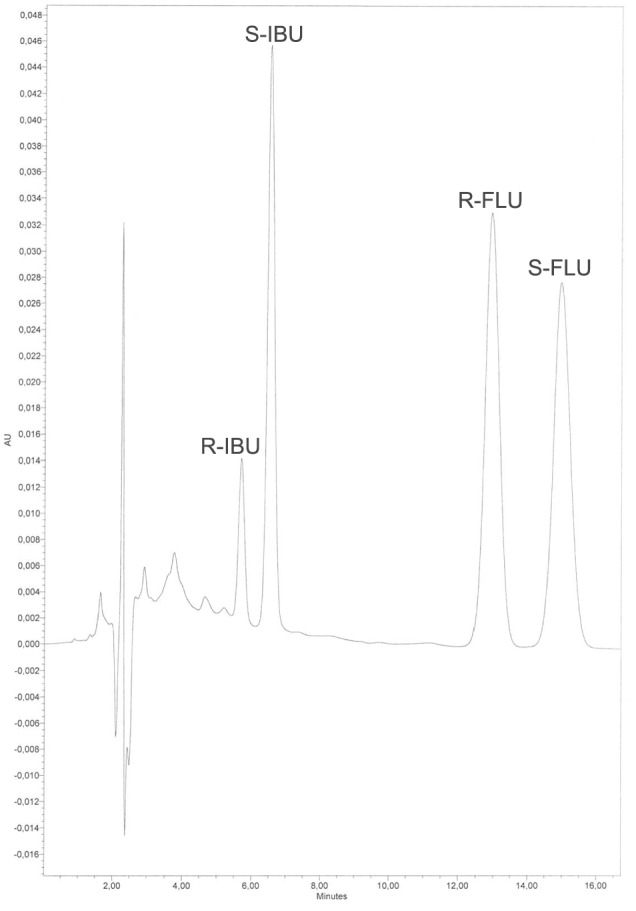
A typical chromatogram of an extract from human plasma. *R*‐Ibuprofen: 7.2 mg L^−1^; *S*‐ibuprofen: 30 mg L^−1^; and *R*/*S*‐flurbiprofen (internal standard): 50 mg L^−1^

The retention times of *R*‐IBU, *S*‐IBU, *R*‐flurbiprofen, and *S*‐flurbiprofen were 5.6, 6.5, 12.7, and 14.7 min, respectively. No interfering peaks were detectable (Figure 1). The calibration curves were linear up to 60 μl ml^−1^, and the coefficient of determination (*r* ^2^) was always >0.99. The coefficient of variations at 0.5, 5, and 30 mg L^−1^ were 12.2%, 2.8%, and 3.1% for *S*‐IBU (*n* = 10), and 11.3%, 3.1%, and 3.2% for *R*‐IBU (*n* = 10), respectively. Recovery reached 91.4% for *S*‐IBU and 91.7% for *R*‐IBU. The limits of detection, defined as a signal‐to‐noise ratio of 3:1, were 0.5 mg L^−1^ for both *S*‐ and *R*‐IBU.

#### 2.1.2 PK analysis

**FIGURE 2:**
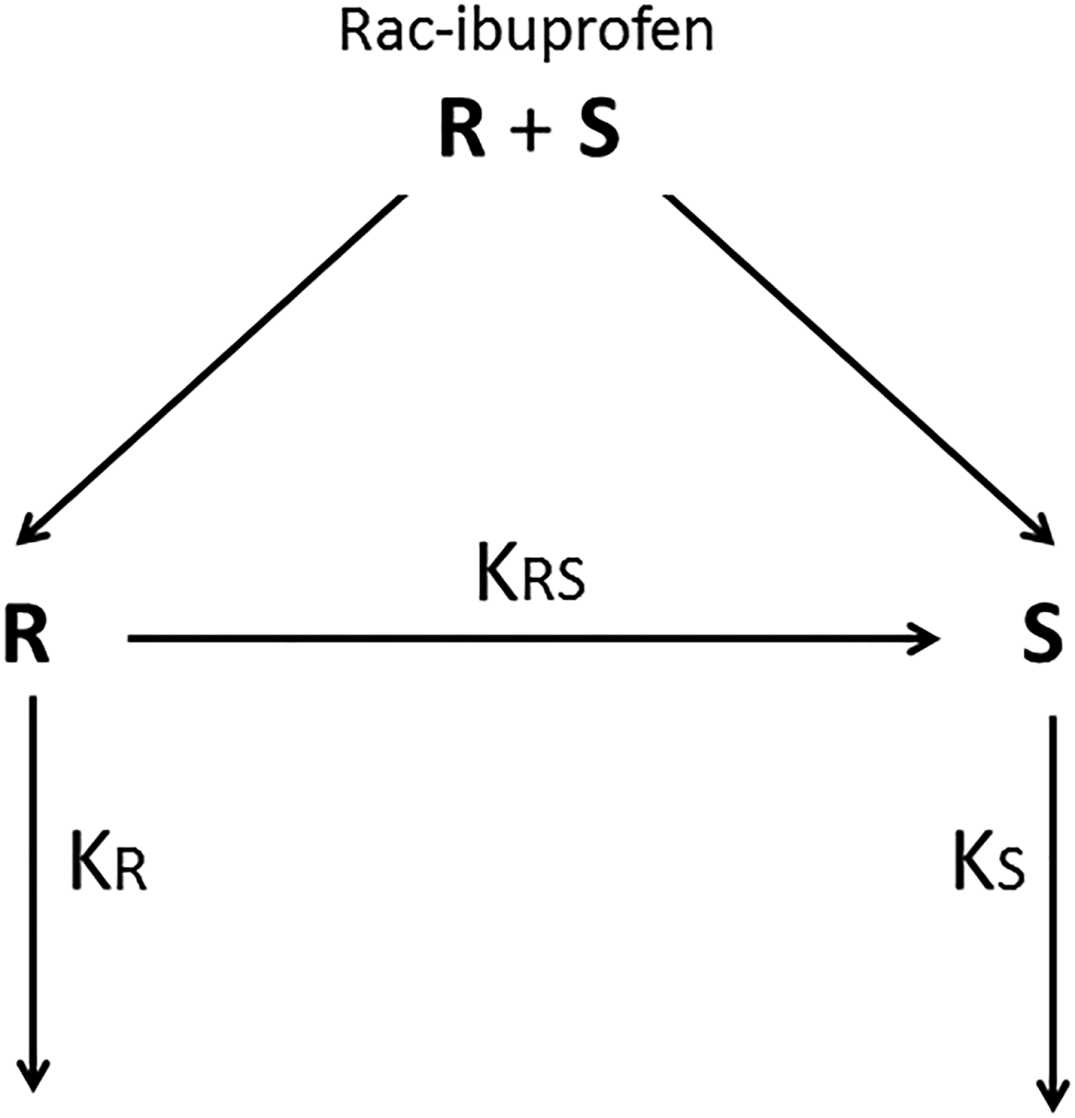
Pharmacokinetic model including rate constants of unidirectional chiral inversion from *R*‐ibuprofen to *S*‐ibuprofen (*K*_*RS*_) and elimination of two enantiomers (*K*_*R*_ and *K*_*S*_)

The time courses of *S*‐IBU and *R*‐IBU plasma concentrations after the first administration were described by a first‐order, one‐compartment open model with different elimination rate constants for *S*‐IBU (*K*_*S*_) and *R*‐IBU (*K*_*R*_), and a unidirectional *R*‐IBU to *S*‐IBU conversion rate constant (*K*_*RS*_) (Figure 2). On these premises, the decay of *R*‐IBU concentrations can be described by two parallel processes (elimination and conversion) according to the following equation:

(1) $$\left[R‐\mathrm{IBU}\right]=R_{0}\times e^{-\left(K_{\mathrm{RS}}+K_{R}\right)\times t},$$

where *R*_0_ is the *R*‐IBU concentration measured at the end of the rac‐IBU infusion, (*K*_*RS*_ + *K*_*R*_) is the overall elimination rate constant, and *t* is time.

Equation 1 was fitted to the *R*‐IBU concentrations measured at 0–6–24 h after the first dose with the best‐fit program of GraphPad 6.0 software, and the rate constant (*K*_*RS*_ + *K*_*R*_) was acquired. If *R*‐IBU concentrations at 24 h were below the limit of detection, the elimination rate constants would be calculated by the slope of the line connecting the log_10_‐concentrations measured at 0 and 6 h: (*K*_*RS*_ + *K*_*R*_) = slope × 2.303.

Then, the following PK parameters were calculated: elimination half‐life (*T*½ = ln(2)/(*K*_*RS*_ + *K*_*R*_)), volume of distribution (VD = dose/kg/*R*_0_), area under the concentration–time curve (AUC = *R*_0_/(*K*_*RS*_ + *K*_*R*_)), and plasma clearance (CL = VD × (*K*_*RS*_ + *K*_*R*_)).

The *S*‐IBU concentration time course, on the other hand, was the result of two opposite processes: *S*‐IBU elimination and *S*‐IBU formation by *R*‐IBU chiral inversion. The elimination process was modeled with a monoexponential equation:

(2) $$\left[S‐\mathrm{IBU}\right]=S_{0}\times e^{-K_{S}\times t},$$

The plasma profile of *S*‐IBU concentrations deriving from *R*‐IBU inversion can be modeled with the equation describing metabolite formation from a parent drug^11^:

(3) $$\left[S‐\mathrm{IBU}\right]=\left[R_{0}\times K_{\mathrm{RS}}/\left(K_{\mathrm{RS}}+K_{R}-K_{S}\right)\right]\times \left(e^{–K_{S}\times t}-e^{–\left(K_{\mathrm{RS}}+K_{R}\right)\times t}\right),$$

where *S*_0_ and *R*_0_ are the concentrations of *S*‐ and *R*‐IBU measured at the end of the rac‐IBU infusion, *K*_*RS*_ is the *R*‐ to *S*‐IBU conversion rate constant, *K*_*R*_ is the *R*‐IBU elimination rate constant, *K*_*S*_ is the elimination rate constant for *S*‐IBU, and *t* is time.

Merging Equation 2 with 3, we obtain the final model describing the *S*‐IBU concentration profile after the first intravenous dose:

(4) $$\left[S‐\mathrm{IBU}\right]=S_{0}\times e^{–K_{S}\times t}+\left[R_{0}\times K_{\mathrm{RS}}/\left(K_{\mathrm{RS}}+K_{R}-K_{S}\right)\right]\times \left(e^{–K_{S}\times t}-e^{–K\left(\mathrm{RS}+R\right)\times t}\right).$$

Equation 4 was fitted to the *S*‐IBU concentrations measured 0, 6, and 24 h after the first dose with the best‐fit program of GraphPad 6.0 software. *S*_0_, *R*_0_, and (*K*_*RS*_ + *K*_*RS*_) were measured experimentally for each subject, so the only unknown variables to be ascertained were *K*_*S*_ and *K*_*RS*_. The last unknown variable, *K*_*R*_, was then obtained by subtracting *K*_*RS*_ from (*K*_*RS*_ + *K*_*R*_). Then, the following PK parameters were calculated: elimination half‐life (*T*½ = ln(2)/*K*_*S*_), volume of distribution (VD = dose/kg/*S*_0_), area under the concentration–time curve (AUC = *S*_0_/*K*_*S*_ + *R*_0_/*K*_*RS*_ − *R*_0_/*K*_*S*_), and plasma clearance (CL = VD × *K*_*S*_).

The fraction of *R*‐IBU converted into *S*‐IBU (*f*) is given by

(5) $$f=K_{\mathrm{RS}}/\left(K_{R}+K_{\mathrm{RS}}\right).$$

Based on the PK parameters obtained after the first rac‐IBU dose, the time courses of the *S*‐ and *R*‐IBU plasma concentrations following repeated doses were simulated using the principle of superposition. Enantiomer plasma concentrations measured at 48 and 72 h after completing the first dose of rac‐IBU were then compared with those predicted by the model.

#### 2.1.3 Statistical analysis

Continuous data were presented as means ± standard deviations (SDs) and ranges of values. The correlation between the demographic or laboratory characteristics and the PK parameters was examined using linear regression analysis, with a significance level of 5%.

## 3 RESULTS

**FIGURE 3:**
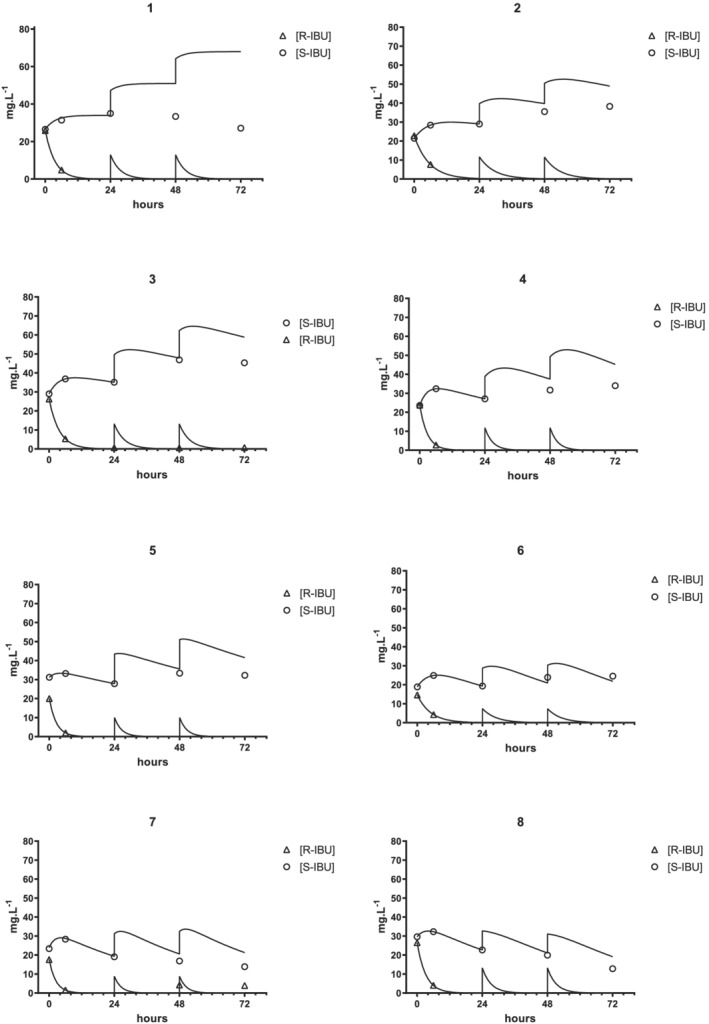
Measured plasma concentrations of *S*‐ibuprofen (circles) and *R*‐ibuprofen (triangles) and curves simulated on the basis of first‐dose best‐fit analyses. Cases 1–8

**FIGURE 4:**
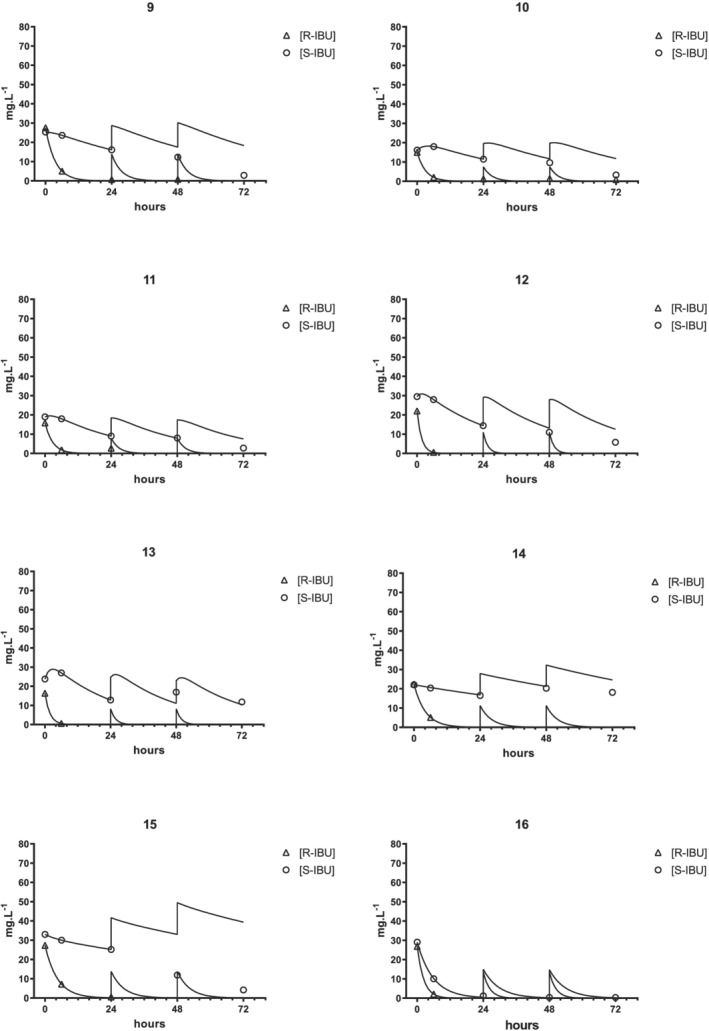
Time courses of plasma concentrations of *S*‐ibuprofen (circles) and *R*‐ibuprofen (triangles) and curves simulated the basis of first‐dose best‐fit analyses. Cases 9–16

PK data were obtained from 16 neonates whose clinical characteristics are listed in Table 1. The time courses of the *S*‐IBU and *R*‐IBU concentrations and the corresponding best‐fit curves and simulations are shown for each subject in Figure 3 (Cases 1–8) and Figure 4 (Cases 9–16).

**TABLE 1:** Demographic and laboratory characteristics at birth

Parameter	Mean	±SD	Range
Birth weight (g)	1186	459	500–2000
Gestational age (weeks)	28.7	2.9	24–32
Age at first dose (h)	58.8	9.8	40–72
Creatinine (mg dl^−1^)	0.82	0.14	0.55–1.10
Aspartate transaminase (U L^−1^)	33.3	10.2	17–50
Alanine transaminase (U L^−1^)	6.6	2.0	3–12
Albumin (g dl^−1^)	2.9	0.46	2.2–3.5
Total bilirubin (mg dl^−1^)	5.1	1.4	3.6–8.6
Conjugated bilirubin (mg dl^−1^)	1.18	1.74	0.44–7.18
Prothrombin time (%)	65.4	6.7	58–75

In 13 of the 16 cases, the *S*‐IBU concentration profiles showed a “hump” at around 6 h (Cases 1–13, Figures 3 and 4), which was attributed to the unidirectional chiral inversion of *R*‐IBU to *S*‐IBU (Equation 4). In 10 of these 13 cases, *S*‐IBU concentrations were higher at 6 h than at the end of the infusion, and in five cases, they remained so even at 24 h. This unusual behavior prompted us to check whether some amounts of *R*‐IBU might be converted into *S*‐IBU after blood sampling. Blank plasma samples spiked with rac‐IBU (10 mg L^−1^) were assayed, kept at 4°C for 24 h, and then assayed again. No differences were noted in the results for either assay, so the possibility of *S*‐IBU forming in vitro after sampling could be ruled out.

In Case 1 (Figure 3), *S*‐IBU concentrations steadily increased up to 24 h. Because *K*_*S*_ was practically nil (=8.1 × 10^−14^), this increase was entirely attributable to the chiral inversion process. In the last three cases (Figure 4, Cases 14–16), the decay in *S*‐IBU concentrations was best described by the monoexponential equation 2, indicating minimal or no chiral inversion.

The PK parameters of *S*‐IBU calculated for each subject are shown in Table 2. The mean values (±SDs) of *T*½, VD, and CL were 41.8 h (±35.0), 207.1 ml kg^−1^ (±44.0), and 7.01 ml h^−1^ kg^−1^ (±7.25), respectively. Linear regression analysis showed that total bilirubin was the only parameter correlating significantly with *S*‐IBU CL (*r* ^2^ = 0.44; *p* = 0.013; positive slope) and *T*½ (*r* ^2^ = 0.37; *p* = 0.027; negative slope). No correlation was found with VD.

**TABLE 2:** *S*‐IBU pharmacokinetic parameters

Cases	*S*_0_ (mg L^−1^)	*K*_*S*_ (h^−1^)	*T*½ (h)	*K*_*RS*_/*K*_*S*_	AUC_*S*_ (mg h L^−1^)	AUC_*R*>*S*_ (mg h L^−1^)	AUC_tot_ (mg h L^−1^)	VD (L kg^−1^)	CL (ml h^−1^ kg^−1^)
1^a^	26.5	‐	‐	‐	‐	‐	‐	188.6	‐
2	21.5	0.0058	119.5	16.22	3701.7	2010.6	5712.3	232.9	1.35
3	29.0	0.0062	112.0	18.70	4684.9	1851.4	6536.3	172.5	1.07
4	23.6	0.0124	55.9	15.32	1901.6	1007.6	2909.3	212.0	2.63
5	31.2	0.0105	65.7	8.32	2959.2	429.7	3388.9	160.3	1.69
6	18.9	0.0224	30.9	8.08	842.9	570.0	1413.6	264.8	5.93
7	23.4	0.0238	29.1	9.42	983.2	408.0	1391.2	213.9	5.09
8	29.7	0.0219	31.7	4.34	1356.2	365.0	1721.2	168.6	3.69
9	25.3	0.0222	31.3	0.88	1143.0	85.9	1228.9	197.3	4.37
10	16.2	0.0274	25.3	4.36	590.4	198.1	788.6	308.8	8.47
11	19.0	0.0402	17.3	2.19	472.7	95.8	568.5	263.4	10.58
12	29.5	0.0370	18.7	3.93	797.0	141.7	938.7	169.5	6.27
13	23.8	0.0422	16.4	8.49	563.4	251.5	814.9	210.3	8.87
14	22.1	0.0113	61.2	‐	1950.6	‐	1950.6	226.2	2.56
15	33.0	0.0834	8.3	‐	395.6	‐	395.6	151.6	12.64
16	29.0	0.1742	4.0	‐	166.7	‐	166.7	172.2	29.99
Mean	25.1	0.0361	41.8	8.35	1500.6	618.0	1995.0	207.1	7.01
SD	4.9	0.0429	35.0	5.75	1325.1	664.1	1899.0	44.0	7.25
CV%	19.4	119.1	83.8	68.9	88.3	107.5	95.2	21.2	103.4
Min	16.2	0.0058	4.0	0.88	166.7	85.9	166.7	151.6	1.07
Max	33.0	0.174	119.5	18.70	4684.9	2010.6	6536.3	308.8	29.99

^a^ The resulting *K*_*S*_ value was extremely low (8.1 × 10^−14^ h^−1^), so the other PK parameters were not calculated, apart from VD.

Simulations of repeated rac‐IBU administrations based on 13 neonates' individual PK parameters showed that *S*‐IBU concentrations at 48 and/or 72 h were lower than predicted, probably due to changes in the clinical condition of the neonates in the first days of life.

Equation 1 was fitted to IBU concentrations measured at 0–6–24 h in five of 16 cases (Cases 3, 9, 10, 11, and 15; Table 3). In the other 11 cases, whose *R*‐IBU concentrations at 24 h fell below the detection limit, the slope of the curves were calculated by the log_10_‐transformed concentrations found at 0 and 6 h (see Section 2). Figures 3 and 4 show only the *R*‐IBU concentrations which were above the detection limit.

**TABLE 3:** *R*‐IBU pharmacokinetic parameters

Cases	*R*_0_ (mg L^−1^)	*K*_*RS*_ + *K*_*R*_ (h^−1^)	*T*½ (h)	*K*_*RS*_ (h^−1^)	*K*_*R*_ (h^−1^)	*R* → *S* (%)	AUC (mg h L^−1^)	VD (L kg^−1^)	CL (ml h^−1^ kg^−1^)
1	25.9	0.280	2.48	0.081	0.199	0.29	95.6	193.3	54.1
2	22.8	0.184	3.77	0.094	0.090	0.51	126.8	219.2	40.3
3	26.3	0.265	2.61	0.116	0.150	0.44	102.4	190.1	50.5
4	23.7	0.361	1.92	0.190	0.171	0.53	68.7	210.6	76.0
5	20.0	0.388	1.79	0.088	0.300	0.23	54.1	250.0	96.9
6	14.6	0.207	3.35	0.181	0.026	0.87	72.5	342.0	70.8
7	17.6	0.407	1.70	0.224	0.183	0.55	45.5	283.6	115.4
8	26.5	0.315	2.20	0.095	0.220	0.30	87.3	188.9	59.5
9	27.6	0.284	2.44	0.020	0.264	0.07	100.7	181.1	51.4
10	15.0	0.330	2.10	0.120	0.210	0.36	47.3	333.6	110.1
11	15.8	0.361	1.92	0.088	0.273	0.24	45.8	316.1	114.1
12	22.0	0.610	1.14	0.145	0.465	0.24	38.8	227.2	138.6
13	16.4	0.553	1.25	0.358	0.195	0.65	31.7	305.1	168.7
14	22.4	0.246	2.82	‐	‐	‐	93.9	223.1	54.9
15	27.3	0.223	3.11	‐	‐	‐	125.7	183.3	40.9
16	26.9	0.426	1.63	‐	‐	‐	66.4	186.2	79.3
Mean	21.9	0.340	2.26	0.138	0.211	0.41	75.2	239.6	82.6
SD	4.7	0.119	0.74	0.085	0.106	0.21	30.4	57.6	37.8
CV%	21.6	34.9	32.6	61.7	50.1	52.8	40.4	24.0	45.7
Min	14.6	0.184	1.14	0.020	0.026	0.07	31.7	181.1	40.3
Max	27.6	0.610	3.77	0.358	0.465	0.87	126.8	342.0	168.7

The related PK parameters of each subject are shown in Table 3. The mean values (±SDs) of *T*½, VD, and CL were 2.26 h (±0.74), 239.6 ml kg^−1^ (±57.6), and 82.6 ml h^−1^ kg^−1^ (±37.8), respectively. Linear regression analysis revealed that nonconjugated bilirubin was the only parameter significantly correlating with *R*‐IBU CL (*r* = 0.61; *p* = 0.021) and *T*½ (*r* = −0.75; *p* = 0.0018). No correlation was found with VD. The fraction of *R*‐IBU converted into *S*‐IBU averaged 0.41, with a wide intersubject variability (range: 0.07–0.87).

## 4 DISCUSSION

On the whole, our results match those of previous studies in preterm neonates reporting a reduced clearance and prolonged *T*½ of rac‐IBU (particularly for *S*‐IBU) compared with adults (Table 4). Some new findings emerged from our study, however. Surprisingly, in 10 of our 16 cases, the *S*‐IBU plasma concentrations increased in the 6 h after ending the infusion of the drug, and in five cases, they remained higher even 24 h later. In another three cases, a slight “hump” appeared during the elimination phase, and in the last three, the *S*‐IBU decay was apparently monoexponential. These mixed findings are probably due to varying combinations of different *R*‐ to *S*‐IBU conversion rates (% chiral inversion: 41 ± 21) and *S*‐IBU elimination rates (*T*½: 41.8 ± 35.0 h). Such PK behavior has never been reported before in adults or children.^12^, ^13^, ^14^, ^15^, ^16^, ^17^, ^18^, ^19^, ^20^, ^21^, ^22^, ^23^ The reported percentages of chiral inversion in the two age groups are similar to those found in our sample (53–65%), but the *R*‐IBU *T*½ is much shorter (about 2 h).^24^ In such kinetic conditions, *S*‐IBU concentration profiles are hardly distinguishable from simple monoexponential decay, even though chiral inversion occurs.

**TABLE 4:** Population characteristics and results of other studies on ibuprofen pharmacokinetics in preterm infants

Reference	No. of subjects	Gestational age (weeks)	Birth weight (g)	Route	Compound assayed	PK analysis	*T*½ (h)	CL (ml h^−1^ kg^−1^)	VD (ml kg^−1^)
Aranda et al.^2^	21	26.8	945	Intravenous	rac‐Ibuprofen	One compartment	30.5	2.06	62.1
Van Overmeire et al.^3^	27	28.6	1250			Two compartments	43.1	9.49	354
Hirt et al.^4^	66	28	1015			One compartment (sparse blood samples)	42.2	9.41	397
Sharma et al.^5^	20	30.5	1262	Oral		One compartment	15.7	‐	‐
Gregoire et al.^8^	108	26.9	880	Intravenous	*S*‐Ibuprofen	One compartment (sparse blood samples)	34.3	3.5	173
					*R*‐Ibuprofen		8.3	25.5	306
Engbers et al.^7^	67	26.1	870	*S*‐Ibuprofen	One compartment (sparse blood samples)	‐	4.6^a^	269 ^a^
					*R*‐Ibuprofen		‐	220 ^a^	352 ^a^
Present study	16	28.7	1186	*S*‐Ibuprofen	One compartment	41.8	7.01	207
					*R*‐Ibuprofen		2.3	82.6	240

^a^ Values estimated for a newborn at a postnatal age of 6 days, a gestational age of 26 weeks, and a body weight of 860 g.

Our study has some analogies with the one by Gregoire et al., who assayed plasma concentrations of both IBU enantiomers after intravenous administration of rac‐IBU (10–5–5 mg kg^−1^) and analyzed their data with the same PK model.^8^ They collected sparse data from three different trials, however, and estimated PK parameters using a population analysis. They did not analyze *S*‐IBU concentration profiles in individual patients, and the average concentration curve they obtained was not convex but nearly linear (Figure 3 in the above‐mentioned study).

Gregoire et al. also reported that the plasma *T*½ of *S*‐IBU did not change (34.3 h) during the first 3 days of life.^8^ Our results differ in this respect, as 13 of 16 neonates had lower *S*‐IBU concentrations on the second and/or third postnatal day than those predicted on the grounds of the first‐day PK parameters (Figures 3 and 4), indicating that *S*‐IBU clearance increased or the volume of distribution decreased or both.

**FIGURE 5:**
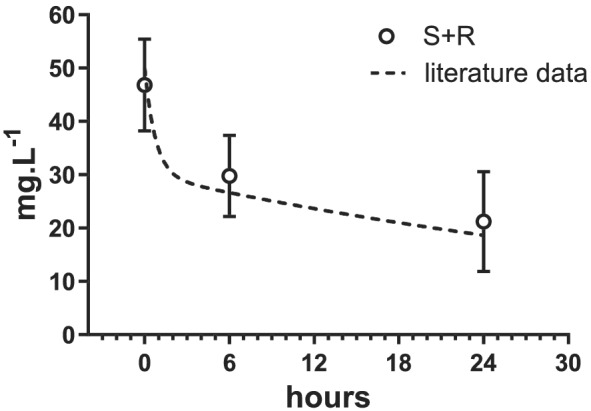
Mean total plasma concentrations (±SDs) of ibuprofen (*S* + *R*) after first dose (open circles), superimposed on concentration profile of rac‐IBU reported by Van Overmeire et al.^3^ (dashed line)

*S*‐IBU elimination depends largely on the activity of cytochrome CYP2C9.^25^, ^26^, ^27^, ^28^ Treluyer et al. reported that CYP2C protein was not expressed in the human fetal liver but rapidly developed during the first week of life.^29^ These changes were paralleled by an increase in CYP2C RNA, driven mainly by CYP2C9 RNA. These in vitro data match our clinical findings of a very long *S*‐IBU *T*½ at birth (41.8 h), followed by lower than predicted *S*‐IBU concentrations after the second and/or third doses of IBU. Van Overmeire et al. likewise reported a significant decrease in *T*½ from the first to the third dose of IBU (from 43.1 to 26.8 h, on average) and a parallel decrease in the AUC and central VD.^3^ They measured total IBU concentrations (*S* + *R*), however, and used a two‐compartment model to describe the concentration time course. It is worth noting that the rac‐IBU concentrations reported by the above authors (Figure 1 in the cited paper) are almost identical to the sums of the *S*‐IBU and *R*‐IBU concentrations found in the present study (Figure 5), indicating that the biexponential model they used did actually describe a rapid elimination of *R*‐IBU (early exponential decay) and slow elimination of *S*‐IBU (late exponential decay).

Further support for the hypothesis of a fast postnatal maturation of IBU metabolism came from Hirt et al., who carried out a population analysis on sparse concentrations of rac‐IBU (*S* + *R*), finding that IBU *T*½ gradually decreased from 42.2 h at 3 days to 9.8 h at 8 days.^4^ Engbers et al. recently implemented an interesting model that correlates *S*‐IBU and *R*‐IBU clearance rates with postnatal and gestational ages.^7^

Our mean estimate of *R*‐IBU *T*½ after the first dose was 2.3 h (a value similar to the ≈2 h seen in adults), whereas Gregoire et al. found a plasma *T*½ for *R*‐IBU of 8.3 h after the first dose, which rapidly dropped to 1.3 h at the third dose.^8^ This discrepancy may be due to difference in postnatal age at the first IBU administration between the two studies, which was 24 h in the study of Gregoire et al. and 40–72 h (58.8 h, on average) in ours.

The mean fraction of *R*‐IBU converted to *S*‐IBU found in our study (0.41) is higher than the one reported by Gregoire et al. (0.17) and slightly lower than in adults (0.53–0.65).^8^, ^24^ It is important to bear in mind that *R*‐IBU unidirectional chiral inversion occurs through three sequential steps catalyzed by one acyl‐CoA synthase (which produces *R*‐IBU‐CoA), one epimerase (which converts *R*‐IBU‐CoA into *S*‐IBU‐CoA), and one hydrolase (which delivers *S*‐IBU).^10^ Acyl‐CoA synthase activity in mouse heart is reportedly 14 times greater at birth than in the embryonic period, and it is 1.4–1.6 times greater in newborn than in adult rabbit skeletal muscle.^30^, ^31^ Although no data are available on the postnatal development of these enzymes in humans, it is reasonable to assume that chiral inversion is well developed in human neonates too.

According to our data, 59% of *R*‐IBU is not converted to *S*‐IBU but cleared by other routes. In vitro studies on liver microsomes indicate that *R*‐IBU is a substrate of CYP2C9 and, to a lesser extent, of CYP2C8.^27^, ^28^ Most clinical studies confirm the dominant role of CYP2C9, but two reports identified CYP2C8 as the main cytochrome responsible for *R*‐IBU elimination.^25^, ^26^, ^32^, ^33^ Whichever cytochrome is involved, it is difficult to explain why *R*‐IBU *T*½ is not prolonged at birth like that of *S*‐IBU, given that the activity of both CYP2C9 and CYP2C8 is depressed in the neonatal liver.^29^ Plasma protein binding of rac‐IBU is lower in neonates (94%) than in adults (98%), but we do not know whether *R*‐IBU binding is selectively reduced, leading to an increase in its clearance.^24^ Other elimination mechanisms, as well as metabolism by cytochromes CYP2C9 and CYP2C8, may be at work in the newborn, and this possibility deserves further investigation.

We also found a positive correlation between IBU enantiomer clearance and total bilirubin (*S*‐IBU) or unconjugated bilirubin (*R*‐IBU) levels. We know that IBU shares the same albumin‐binding site as bilirubin and that IBU clearance depends heavily on protein binding (low liver extraction), so it may be that high bilirubin concentrations displace IBU enantiomers from their binding site, thus increasing their clearance.^34^ Clearly, this hypothesis will also require further investigation.

The main limitation of our study concerns the small number of plasma concentrations on which the analysis was based. There are two reasons for this: (i) ethical considerations prevented us from taking more blood samples from low‐weight, fragile newborns, and (ii) our original aim was not to perform a detailed PK analysis of IBU enantiomers but to assess drug exposure and possible correlations with the PDA closure rate. The sole purpose of the sampling planned at 6 h after rac‐IBU infusion was to keep clinicians blind to the drug used in each neonate (because paracetamol was administered every 6 h). A posteriori, this sampling time proved very important in revealing the extent of chiral inversion and prompted us to identify the appropriate PK model for describing the *S*‐IBU plasma profile. From a strictly mathematical standpoint, at least three concentrations are needed to calculate the two variables of the model (*K*_*RS*_ and *K*_*S*_). Although more data would have yielded more accurate estimates of the PK parameters, the *S*‐IBU and *R*‐IBU *T*½ values that we obtained substantially match those reported by other authors in preterm neonates with PDA.^2^, ^3^, ^4^, ^5^, ^7^, ^8^

## 5 CONCLUSIONS

Our study confirmed that *S*‐IBU elimination is markedly slower in premature newborn than in adults and tends to accelerate over the first days of life. We also found that the rate of chiral inversion from *R*‐ to *S*‐IBU at birth varies considerably and may be responsible for an odd increase in *S*‐IBU plasma concentrations after completing the drug's infusion, which persists even after 24 h in some cases. This evidence did not emerge from studies based on sparse blood sampling and population analysis.^7^, ^8^ Because *S*‐IBU is much more active than *R*‐IBU, this “additional dose” of *S*‐IBU deriving from chiral inversion may have clinical consequences.

## AUTHOR CONTRIBUTIONS

Study conception and design: P.L., A.C.F., and R.P.; data acquisition: C.A., D.N., G.D.R., S.S., and L.B.; data analysis and interpretation and drafting of manuscript: R.P. All authors revised the manuscript and approved the final version.

## Data Availability

Data are available on request from the authors.
